# Quality of prenatal care questionnaire: instrument development and testing

**DOI:** 10.1186/1471-2393-14-188

**Published:** 2014-06-03

**Authors:** Maureen I Heaman, Wendy A Sword, Noori Akhtar-Danesh, Amanda Bradford, Suzanne Tough, Patricia A Janssen, David C Young, Dawn A Kingston, Eileen K Hutton, Michael E Helewa

**Affiliations:** 1 College of Nursing and Departments of Community Health Sciences and Obstetrics, Gynecology and Reproductive Sciences, College of Medicine, Faculty of Health Sciences, University of Manitoba, 89 Curry Place, Winnipeg R3T 2N2, Manitoba, Canada; 2 School of Nursing and Department of Clinical Epidemiology and Biostatistics, Faculty of Health Sciences, McMaster University, 1280 Main Street West, Hamilton L8S 4K1, Ontario, Canada; 3 Gilbrea Centre for Studies in Aging, McMaster University, 1280 Main Street West, Hamilton L8S 4M4, Ontario, Canada; 4 Departments of Paediatrics and Community Health Sciences, Faculty of Medicine, University of Calgary, 2888 Shaganappi Trail NW, Calgary T3B 6A8, Alberta, Canada; 5 School of Population and Public Health, University of British Columbia, 2206 East Mall, Vancouver V6N 1Z3, British Columbia, Canada; 6 Department of Obstetrics and Gynecology, IWK Health Centre, Dalhousie University, 5980 University Avenue, P.O. Box 9700, Halifax B3K 6R8, Nova Scotia, Canada; 7 Faculty of Nursing, University of Alberta, 5-258 Edmonton Clinic Health Academy, 11405-87th Avenue, Edmonton T6G 1C9, Alberta, Canada; 8 Department of Obstetrics and Gynecology and Department of Clinical Epidemiology and Biostatistics, Faculty of Health Sciences, McMaster University, 1280 Main Street West, Hamilton L8S 4K, Ontario, Canada; 9 Department of Obstetrics, Gynecology and Reproductive Sciences, College of Medicine, Faculty of Health Sciences, University of Manitoba, 735 Notre Dame Avenue, University of Manitoba, Winnipeg R3T 2N2, Manitoba, Canada

**Keywords:** Prenatal care, Quality of care, Measurement, Instrument, Reliability, Validity, Psychometric testing

## Abstract

**Background:**

Utilization indices exist to measure quantity of prenatal care, but currently there is no published instrument to assess quality of prenatal care. The purpose of this study was to develop and test a new instrument, the Quality of Prenatal Care Questionnaire (QPCQ).

**Methods:**

Data for this instrument development study were collected in five Canadian cities. Items for the QPCQ were generated through interviews with 40 pregnant women and 40 health care providers and a review of prenatal care guidelines, followed by assessment of content validity and rating of importance of items. The preliminary 100-item QPCQ was administered to 422 postpartum women to conduct item reduction using exploratory factor analysis. The final 46-item version of the QPCQ was then administered to another 422 postpartum women to establish its construct validity, and internal consistency and test-retest reliability.

**Results:**

Exploratory factor analysis reduced the QPCQ to 46 items, factored into 6 subscales, which subsequently were validated by confirmatory factor analysis. Construct validity was also demonstrated using a hypothesis testing approach; there was a significant positive association between women’s ratings of the quality of prenatal care and their satisfaction with care (*r* = 0.81). Convergent validity was demonstrated by a significant positive correlation (*r* = 0.63) between the “Support and Respect” subscale of the QPCQ and the “Respectfulness/Emotional Support” subscale of the Prenatal Interpersonal Processes of Care instrument. The overall QPCQ had acceptable internal consistency reliability (Cronbach’s alpha = 0.96), as did each of the subscales. The test-retest reliability result (Intra-class correlation coefficient = 0.88) indicated stability of the instrument on repeat administration approximately one week later. Temporal stability testing confirmed that women’s ratings of their quality of prenatal care did not change as a result of giving birth or between the early postpartum period and 4 to 6 weeks postpartum.

**Conclusion:**

The QPCQ is a valid and reliable instrument that will be useful in future research as an outcome measure to compare quality of care across geographic regions, populations, and service delivery models, and to assess the relationship between quality of care and maternal and infant health outcomes.

## Background

The evidence for the effectiveness of prenatal care remains equivocal, despite its widespread use [1,2], and substantial amounts of health care resources “continue to be expended on a tradition of care that has not proven itself equal to the perinatal health issues of today” [3]. Previous research has frequently relied on prenatal care utilization indices to study the association between adequacy of prenatal care and pregnancy outcomes [4-6]; however these indices focus solely on quantifying the use of care and do not adequately assess the content or quality of care [1]. Several studies have highlighted the potential importance of content and quality of care [7-14]. In fact, the “role of adequate utilization has more recently been downplayed and greater credence has been given to the importance of the content, comprehensiveness, and quality of prenatal care” [1].

The content and quality of prenatal care have been measured in different ways. For example, Beeckman and colleagues recently developed the Content and Timing of Care in Pregnancy (CTP) tool to assess women’s receipt of recommended content based on recommendations in national and international guidelines [8]. Participants recorded the timing and content of prenatal care using diaries. These investigators concluded the content items need further refinement prior to larger scale testing of the new measure [8]. Content has also been measured in studies that examined the effect of adherence to recommended prenatal care content, assessed from medical records, on pregnancy outcomes [9-11]. Other studies have investigated the impact of enhanced or augmented prenatal services [12,13,15] or new models of care, such as group prenatal care [16], on outcomes. The quality of prenatal care has been evaluated using focus groups to explore quality as experienced by women [17-19], developing audit indicators of quality of prenatal care [20], or using checklists, observations and exit interviews [21]. Wong and colleagues developed an instrument to measure the quality of interpersonal processes of care [22], but this instrument measures only one dimension of quality. To date, research on the effectiveness of prenatal care has been hindered by the lack of an instrument that comprehensively measures quality of prenatal care.

Assessment of prenatal care has focused primarily on women’s satisfaction, but often without clear distinction between the constructs of satisfaction and quality of care. Research to empirically test the relationships between these variables provides evidence that perceived quality affects satisfaction with health care, and that quality of care and consumer satisfaction are distinct constructs [23,24]. Quality is defined as a judgment or evaluation of several dimensions specific to the service being delivered, whereas satisfaction is an affective or emotional response to a specific consumer experience [23,24]. Satisfaction measures tend to include components that are considered elements of quality, such as structure of service delivery (wait time, continuity of care, physical environment) and process of care (advice received, explanations given by care provider, technical quality of care) [25-27]. These instruments have limitations in that they do not discriminate between quantity and quality of care [28], generally lack psychometric evaluation [27], and do not adequately tap varying dimensions of the uniqueness of prenatal care [27]. Finally, satisfaction measures are insensitive, as most women report high levels of satisfaction with prenatal care [25,26], particularly when measured after delivery [29].

Approaches to the assessment of quality of prenatal care have been largely atheoretical. Among the few studies that have based their selection of measures on a theoretical framework [21,30-32], the two frameworks most commonly used were Donabedian’s [33,34] model of quality and Aday and Andersen’s [35,36] theoretical framework for the study of access to medical care. The latter model is primarily focused on health service utilization issues. There is a need to develop a theoretically-grounded measure of prenatal care quality that is distinct from satisfaction measures in order to better evaluate the relationship between quality of prenatal care and pregnancy outcomes. The conceptual framework guiding this research was Donabedian’s systems-based model of quality health care [34]. The framework encompasses a three-part approach to quality assessment, in which “good structure increases the likelihood of good process, and good process increases the likelihood of a good outcome” [34]. Structure includes attributes of the setting in which care is provided, such as material and human resources and organizational structure [34]. The process component reflects the actual care given. There are two processes of care: clinical or technical, and interpersonal [37]. According to Donabedian, the goodness of technical performance should be judged in comparison with best practice, while interpersonal process is the vehicle by which technical care is implemented and includes information exchange, privacy, informed choice, and sensitivity [34].

In keeping with the findings of qualitative studies that demonstrated the value women place on the interpersonal processes of prenatal care (including communication, decision-making and interpersonal style), recent attention has been focused on the conceptualization of these processes, their measurement, and their impact on women’s satisfaction and perception of quality of care [7,22]. Research has demonstrated that ineffective communication is a barrier to prenatal care utilization [38-40]. Care provider characteristics, such as lack of perceived concern and respect, being task focused and conveying an authoritarian approach, also deter use of prenatal care [40-42]. These characteristics also can be a barrier to women disclosing health concerns [43]. Thus interpersonal processes are important in keeping women engaged in prenatal care and, ultimately, in enhancing outcomes.

The development of an instrument to measure quality of prenatal care can be informed by multiple sources, including the available research evidence regarding effective clinical practices and the perspectives of care providers and women [21,37]. Because quality of care is determined by the structure of service delivery and service-giving processes [34,44], it encompasses content dimensions through its attention to the technical (e.g., physical examinations and tests) and interpersonal (e.g., health promotion counseling) aspects of care. Care providers are best positioned to comment on clinical aspects of care [21], including that which is knowledge-based but does not necessarily have scientific evidence of effectiveness [37]. Few studies have considered the perspectives of pregnant women in the development of measurement instruments [26,27], and only one tool incorporated both women’s and health care providers’ perspectives [45].

### Purpose and aims of the study

The development of a valid and reliable instrument to measure prenatal care quality is a critical scientific foundation for research to monitor the provision and benefits of prenatal health care services. Donabedian states that consumers make an indispensable contribution to defining and evaluating the quality of care [15]. The purpose of this study was to develop and test a new instrument, the Quality of Prenatal Care Questionnaire (QPCQ), to be completed by consumers (women receiving prenatal care). Specific aims were:

1. To generate items for the QPCQ;

2. To conduct content and face validity assessment and exploratory factor analysis of the QPCQ to determine final items; and

3. To conduct psychometric testing of the final version of the QPCQ.

## Methods

**Figure 1:**
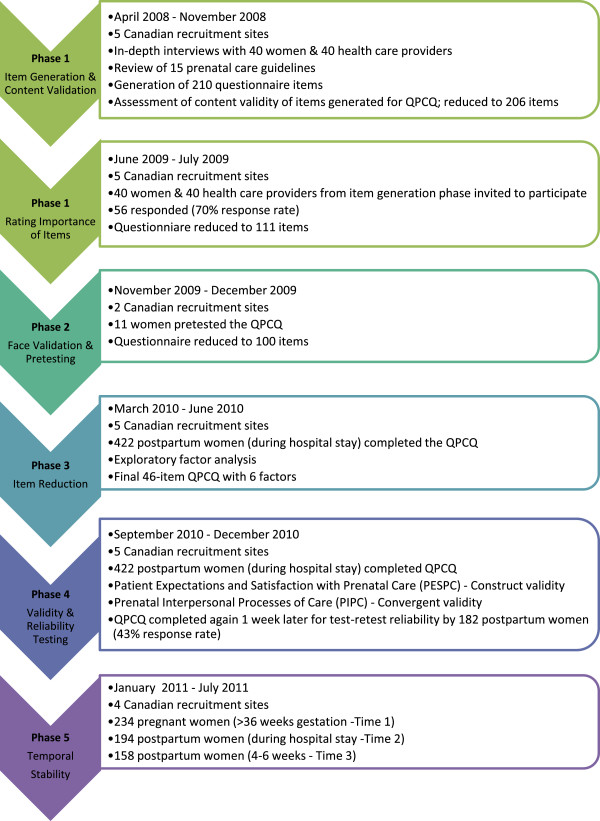
Flow chart of five phases of development and testing of the QPCQ.

This study addressed the development, validation, and evaluation of a research instrument. Guided by the methodological frameworks for developing measurement scales described by Streiner and Norman [46] and Pett, Lackey and Sullivan [47], the study consisted of five phases implemented over the course of 4 years. Refer to Figure 1 for a flow chart of the five phases. Phase One was development of an instrument to measure quality of prenatal care, and included item generation, content validity, rating of importance of items, and item presentation. Phase Two consisted of face validation and pretesting. Phase Three was item reduction using factor analysis. Phase Four involved instrument evaluation, that is, psychometric testing to establish its construct validity, internal consistency reliability, and test-retest reliability. Phase Five involved temporal stability testing. Ethical approval for this study was received from Hamilton Health Sciences/McMaster University Faculty of Health Sciences Research Ethics Board, the University of Manitoba Education/Nursing Research Ethics Board, the University of Calgary Conjoint Health Research Ethics Board, the IWK Health Centre Research Ethics Board, and the University of British Columbia Clinical Research Ethics Board.

### Phase one: item generation, content validation, rating of importance of items, and item presentation

#### Item generation

The first step of the instrument development process was to generate a comprehensive list of items to represent the various components of the construct *quality of prenatal care*. The items for the initial questionnaire were generated from two sources. The first source was a qualitative descriptive study involving in-depth semi-structured interviews with 40 pregnant women and 40 prenatal care providers from five urban centers across Canada (Vancouver, Calgary, Winnipeg, Hamilton, and Halifax), conducted between April and November 2008. The qualitative descriptive study is described in detail elsewhere [48]. In keeping with Donabedian’s suggestion that the goodness of clinical or technical performance should be judged in comparison with best practice [34], the second source of items was a review of the evidence from 15 international guidelines that inform the provision of prenatal care. Table 1 presents a list of the prenatal care guidelines reviewed.

**Table 1:** Prenatal care guidelines reviewed to generate items for the QPCQ based on “A” grade evidence

**Organization name**	**Guideline title**	**Publication date**
The American College of Obstetricians and Gynecologists & American Academy of Pediatrics	Guidelines for Perinatal Care (6th edition)	October 2007
The American College of Obstetricians and Gynecologists	Committee Opinion-Psychological Risk Factors: Perinatal Screening and Intervention	August 2006
The Society of Obstetricians and Gynaecologists of Canada	Healthy Beginnings: Guidelines for Care During Pregnancy and Childbirth	December 1998
	Fetal Health Surveillance: Antepartum and Intrapartum Consensus Guideline	September 2007
Public Health Agency of Canada	Family-Centered Maternity & Newborn Care: National Guidelines	2000
National Institute for Health and Clinical Excellence	Antenatal Care: Routine care for healthy pregnant women	March 2008
The Royal Australian and New Zealand College of Obstetricians and Gynaecologists	Obstetricians and childbirth responsibilities	July 2007
	Prenatal screening for trisomy 21, trisomy 18 and neural tube defects	July 2007
	Mineral and vitamin supplementation in pregnancy	July 2008
	Antenatal screening tests	June 2008
	Diagnosis of Gestational Diabetes Mellitus	June 2008
	Guidelines for the use of Rhd immunoglobulin in Obstetrics in Australia	March 2007
Royal College of Obstetricians and Gynaecologists	Clinical Standards: Advice on Planning the Service in Obstetrics and Gynaecology	July 2002
World Health Organization	What is the effectiveness of antenatal care? (Supplement)	December 2005
	New WHO antenatal care model	2002

#### Rating importance of items

A clinimetric or “clinical sensibility” approach was used to select which of the 206 items in the QPCQ would be retained for the next step of instrument development [49]. This approach relied on the judgments of patients and clinicians rather than on mathematical (psychometric) techniques to determine which items to include [50]. The sample of 40 women and 40 health care providers who participated in the qualitative descriptive study [48] were mailed a copy of the 206-item instrument along with a cover letter and self-addressed, stamped envelope for return in June and July of 2009. Four randomly generated versions of the list of QPCQ items were prepared to avoid response fatigue toward the end of rating all the items. To maximize response rate, a modification of Dillman’s tailored design method was utilized, including a reminder letter and second mailing of surveys to respondents [51]. In the cover letter, participants were given the following instructions: *“When you rate the items, we are not asking you to reflect on your own experiences with prenatal care. Rather, we would like you to rate how important you think each item is in the care provided by health care professionals to pregnant women using a 7-point rating scale from 1 (not very important) to 7 (extremely important).”* Data for this phase were entered into Microsoft Excel. A mean rating score was generated for each item.

#### Item presentation

Once the most important items were selected for inclusion in the QPCQ, the research team discussed and made decisions regarding instrument format, printed layout, wording of instructions to the subjects, wording and structuring of the items, and response format [47]. Our intent was to develop an instrument suitable for self-administration to pregnant or postpartum women.

### Phase two: face validation and pretesting

Once the newly formed instrument had been drafted, it was assessed for face validity and pretested. Face validity refers to the appearance of the instrument to a layperson, and whether the instrument appears to measure the construct [52]. Pretesting was used to ensure that items were clearly written and were being interpreted correctly [46]. Research assistants administered the 111- item version of the QPCQ to 11 pregnant women in two sites (Winnipeg and Hamilton) between November and December 2009 in a location of the participants’ choice (e.g., prenatal care facility, own home). Women were instructed to respond to each item as if they were actually participating in a study, but to mark items that were difficult to read or confusing. The length of time to complete the QPCQ was recorded. Women were then asked a series of questions by the research assistant about the clarity of the instructions and the items, whether the items appear to be related to the construct of quality of prenatal care, suggestions for alternate wording, items that should be added or removed, and the overall appearance of the instrument. The feedback regarding the quality of prenatal care instrument was discussed by the researchers and revisions were made accordingly.

### Phase three: item reduction using exploratory factor analysis

The purpose of this step was to further reduce the number of items in the QPCQ by eliminating any that were redundant or not congruent with the overall construct being measured. We aimed to recruit a convenience sample of at least 400 women (approximately 80 women per study site) to participate in the item reduction step. A sample size of 400 women was determined to be sufficient as Devillis [53] suggests that a sample size of 200 is adequate in most cases of factor analysis, while Comrey and Lee state that a sample size of 300 is good and 500 is very good [54].

#### Setting and sample

Subjects were recruited from hospitals providing obstetrical services in each study site. These hospitals included BC Women’s Hospital, Vancouver, BC; Foothills Hospital, Calgary, AB; St. Boniface General Hospital and Health Sciences Centre Women’s Hospital, Winnipeg, MB; St. Joseph’s Healthcare, Hamilton, ON; and IWK Health Centre, Halifax, NS. Women were eligible to participate if they had given birth to a singleton live infant, were 16 years of age or older, had at least 3 prenatal care visits, and could read and write English. We excluded women with a known psychiatric disorder that precluded participation in data collection, and women who had a stillbirth or early neonatal death because it would be inappropriate to collect data from these women during the grieving process.

#### Recruitment and data collection procedure

Nursing staff of the postpartum units were asked to identify women who met the inclusion criteria and determine their willingness to learn more about the study. Women were then approached by the site research assistant (Vancouver, Calgary, Winnipeg, Halifax) or the research coordinator (Hamilton), who provided a verbal explanation and written information about the study. Signed, informed consent was obtained from those who agreed to participate. Participants completed the QPCQ and a brief demographic form, and received a $20 gift certificate in appreciation for their time and contribution to the study. Data collection for Phase Three was conducted between March and June 2010.

#### Data analysis

Exploratory factor analysis was conducted using SPSS Version 18.0. Exploratory factor analysis is used when the researcher does not know how many factors are needed to explain the interrelationships among a set of items, indicators, or characteristics [47]. This analytic approach involves a series of structure-analyzing procedures to identify the interrelationships among a large set of observed variables and group the variables into dimensions or factors that have similar characteristics [47]. First, a correlation matrix was constructed to summarize the interrelationships among the items in the scale [47]. The matrix was examined to identify any items that were either too highly correlated (*r* ≥ 0.80) or not correlated sufficiently with one another (*r* <0.30), and these items were dropped from the analysis. Exploratory factor analysis was then used to explore the underlying dimensions of the construct of interest [47], since the conceptual framework did not clearly specify a set number of subconcepts or process of care dimensions [55,56]. Principal axis factoring was used to extract the factors, followed by oblique rotation using the direct oblimin procedure [55]. We chose oblique rotation because we did not expect the dimensions to be orthogonal, i.e., uncorrelated with one another. A factor pattern matrix was generated, which contained the loadings that represented the unique relationship of each item to a factor, after controlling for the correlation among the factors [47]. Items with weak loadings (less than 0.40) or that did not load reasonably on any factor were deleted.

### Phase four: validity and reliability testing

Phase Four involved administering the newly designed 46-item QPCQ to women to establish its construct validity, internal consistency reliability, and test-retest reliability. Similarly to the previous phase, participants were recruited from hospital postpartum units in each study site using the same eligibility/ineligibility criteria and recruitment procedure. Study participants were asked to complete a brief demographic questionnaire, the 46-item QPCQ, the Patient Expectations and Satisfaction with Prenatal Care Instrument (PESPC) [27], and the Prenatal Interpersonal Processes of Care (PIPC) instrument [22]. Women were given a second copy of the QPCQ to be completed 1 week later and returned in a stamped self-addressed envelope. Each participant received a $20 gift certificate in appreciation for their time and contribution to the study. Data collection for Phase Four was conducted between September and December 2010.

#### Construct validity

Validity testing of an instrument is on an ongoing process to determine whether there is sufficient evidence to support that it accurately measures the construct it was designed to measure, and the degree to which it performs according to theoretical predictions [57]. First, confirmatory factor analysis was conducted, using the Amos version 7 statistical analysis program, to test the utility of the underlying dimensions of the construct that were previously identified though exploratory factor analysis [47]. A second approach to determining construct validity was through hypothesis testing. According to Donabedian, patient satisfaction is one of the desired outcomes of quality of care [34]. Although different definitions of quality were used, a randomized controlled trial [12] and a cross-sectional study [7] found that women who received “high quality” prenatal care were significantly more likely to be satisfied with their care. We hypothesized that women who rated the quality of their prenatal care higher would have higher ratings of satisfaction with prenatal care. The Pearson correlation between the total QPCQ score and the satisfaction subscale score of the Patient Expectations and Satisfaction with Prenatal Care instrument (PESPC) [27] was estimated. The PESPC is a 41-item self-administered questionnaire designed to measure pregnant women’s expectations and satisfaction with the prenatal care they anticipated and received. The PESPC is structurally valid, and the satisfaction subscale demonstrates an acceptable level of internal consistency (Cronbach’s alpha of 0.94). The third approach was to test the convergent validity principle, whereby different measures of the same construct should correlate highly with each other [52]. Although there is no other instrument that measures quality prenatal care in all its dimensions, one instrument has been developed to measure the quality of interpersonal processes of prenatal care, known as the Prenatal Interpersonal Processes of Care (PIPC) [22]. The PIPC has seven subscales and 30 items that reflect three underlying dimensions: Communication, Patient-Centered Decision Making, and Interpersonal Style. The majority of the seven subscales have acceptable internal consistency reliability (ranging from 0.66 to 0.85) and preliminary evidence of construct validity has been established. It was anticipated that one or more of the PIPC subscales (such as respectfulness/emotional support) would measure similar constructs as one or more of the QPCQ subscales, and if so, the Pearson correlation between the subscales would be estimated.

#### Reliability

Reliability of an instrument is the degree of consistency with which it measures the attribute it is intended to measure [58]. Both internal consistency reliability and test-retest reliability of the QPCQ were assessed.

Internal consistency is based on the average correlation among items within a test [59] and assesses homogeneity or the extent to which all items measure the same construct [58]. Cronbach’s alpha was used to assess the extent to which performance of any one item on the instrument was a good indicator of performance of any other item on the same instrument [57], and was calculated for both the overall scale and each of the subscales. A Cronbach’s alpha coefficient of at least 0.70 is considered acceptable, while 0.80 or greater is desirable [46,59]. In addition, item-to-total scale correlation coefficients for the instrument subscales were examined, as well as whether the Cronbach’s alpha increased if any of the items were deleted.

The test-retest method is a test of stability to determine whether the same results are obtained on repeat administration of the instrument to the same sample. As mentioned previously, women participating in this phase of the study were given a second copy of the QPCQ to be completed one week later and returned by mail. This time interval is within the recommended retest interval of 2 to 14 days [46]. For each participant who returned the second questionnaire, their scores on the QPCQ were summed for time one and time two, and the level of agreement between the two sets of scores was determined using the intra-class correlation coefficient (ICC). Reliability coefficients above 0.70 are considered acceptable [58]. For the sample size calculation, the minimal acceptable level of ICC was set at 0.75 and the upper limit of ICC at 0.85, with α = 0.05 and β = 0.20. Using the method suggested by Walter, Eliasziw and Donner [60], a minimum sample size of 79 subjects was needed.

### Phase five: temporal stability testing

This phase was conducted to assess whether or not women’s responses to the QPCQ were stable between late pregnancy and the postpartum period, in order to determine whether or not the birth experience and outcome might have influenced women’s recall of quality of care and their responses to the questionnaire. This information is needed to inform timing of administration of the questionnaire in future research.

For this phase of the study, we collected data from 234 women in four of the study sites. Women were asked to provide background information and complete a package of questionnaires shortly before they gave birth (after 36 weeks gestation) (Time 1), again during their postpartum hospital stay (Time 2), and then again 4 to 6 weeks after the baby was born (Time 3). Data collection was conducted between January and July 2011. Mean scores on the total QPCQ and each of the subscales were calculated. At first, we used a randomized block design (RBD) analysis of variance to evaluate the differences between the three time points. RBD was used to adjust for the correlations between time points for the same individuals. However, because of an imbalance in the number of participants at different time points and to use the most information available in the data, we followed RBD with conducting a paired t-test between each two time points (i.e., Time 1 and Time 2, Time 1 and Time 3, Time 2 and Time 3). The intra-class correlation coefficient (ICC) was used to examine stability of the QPCQ total score and subscale scores across the three time periods.

## Results

### Phase one: item generation, content validation, rating of importance of items, and item presentation

Results from the qualitative descriptive study [48] and the review of prenatal care guidelines were used to create a blueprint to establish the specific scope and emphasis of our instrument to measure quality of prenatal care, including the major domains to be assessed [52]. The Co-Principal Investigators (MIH & WAS) generated an initial list of 210 items for the preliminary version of the Quality of Prenatal Care Questionnaire (QPCQ). Several of the items were generated from the interview data that informed the development of themes. These themes were organized into three main categories informed by the structure and process components of Donabedian’s [34] model of quality health care. Structure of care themes included access to care, staff and provider characteristics, and the physical setting. Themes under clinical care processes included screening and assessment, health promotion and illness prevention, continuity of care, information sharing, women-centeredness, and non-medicalization of pregnancy. Themes concerning interpersonal care processes included emotional support, approachable interaction style, taking time, and respectful attitude [48]. Items generated from the guideline review reflected components of prenatal care rated as having a high certainty of net benefit (i.e., “A” grade evidence) [61]). The research team then met to review and discuss the list of 210 items, and as the content experts, assessed the content validity of the QPCQ by evaluating each item for its relevance and clarity, and for any repetition of items. Four items judged to duplicate other items were removed.

Ratings of the importance of the 206 items for the QPCQ were received from 56 participants (70% response rate). The overall top 100 items that were rated as most important were retained for the next version of the instrument; these items had a mean rating of 5.7 or higher on a scale of 1 to 7. In order to ensure that the perspectives of women and health care providers were equally represented, we also added any items ranked in the top 50 from either women or providers that were not in the overall top 100. Because there was generally good congruence between women and providers in rating the importance of items, this resulted in only 3 items with high ratings from health care providers and 2 items from women being added to the top 100 items. Six items derived from A-level evidence but not in the top 100 items were also retained. These steps resulted in a QPCQ with 111 items.

When constructing the QPCQ, the research team decided that each item would be rated using a Likert scale with five response categories consisting of “Strongly Disagree” (1), “Disagree” (2), “Neither Agree Nor Disagree” (3), “Agree” (4) and “Strongly Agree” (5). All points on the scale were labeled to prevent the tendency for respondents to endorse labeled points more often when only some are labeled [46]. A selection of items was “reversed” to reduce responder bias that may occur when all items are written as positive [46]. The 111 items were then formatted into the initial version of the QPCQ with the following instructions: *“This questionnaire asks about the prenatal care you received from a physician, midwife, or other health care providers during your pregnancy. You might have seen more than one health care provider for your care but please think of the prenatal care you received overall when completing this questionnaire. Please read each statement carefully and indicate how much you agree or disagree with it by circling the appropriate number.”*

### Phase two: face validation and pretesting

During the pretesting phase, the mean length of time for women to complete the 111-item version of the QPCQ ranged from 10 to 23 minutes, with a mean of 16 minutes. Women indicated that the QPCQ was easy to complete, and only a few items were identified as potentially problematic. Based on this feedback, 11 items were removed from the QPCQ, either because the item was too vague (e.g., “My prenatal care provider was thorough”) or the item was not universally applicable to all pregnant women (e.g., “My prenatal care provider took time to answer my partner’s/family member’s questions”). This resulted in a 100-item questionnaire. In addition, four items underwent wording changes to improve their clarity or completeness (e.g., The item “I fully understood the reasons for tests my prenatal care provider (s) ordered for me” was changed to “I fully understood the reasons for blood work and other tests my prenatal care provider (s) ordered for me”).

### Phase three: item reduction using exploratory factor analysis

The final sample for Phase Three consisted of 422 participants. Demographic characteristics of the participants are summarized in Table 2; cases with missing data on each item were excluded from the analyses. Use of exploratory factor analysis extracted 5-, 6- and 7-factor solutions. The researchers examined the 3 solutions, and selected the 6-factor solution because the items were judged to be the most relevant and grouped into factors in the most meaningful way based on our clinical knowledge and experience. The 6-factor solution reduced the QPCQ to 46 items. These final factors or dimensions comprised the subscales of the QPCQ; the research team met to agree on the names to be assigned to each factor. The six factors are as follows:

**Table 2:** **Demographic characteristics of participants in phases three, four, and five**^**1**^

**Characteristic**	**Phase three**	**Phase four**	**Phase five**
	**Item reduction**	**Validity & reliability**	**Temporal stability**
	**N = 422**	**N = 422**	**N = 234**
	**n (%)**	**n (%)**	**n (%)**
**Recruitment Site**			
Vancouver	82 (19.4)	64 (15.2)	9 (3.8)
Calgary	98 (23.2)	61 (14.5)	79 (33.8)
Winnipeg	77 (18.3)	112 (26.5)	67 (28.6)
Hamilton	86 (20.4)	106 (25.1)	79 (33.8)
Halifax	79 (18.7)	79 (18.7)	0*
**Marital Status**			
Married	281 (66.6)	284 (67.3)	168 (70.9)
Common-law	49 (11.6)	74 (17.5)	35 (14.8)
Living with a partner	10 (2.4)	15 (3.6)	13 (5.5)
Single (never married)	30 (7.1)	45 (10.7)	16 (6.8)
Separated or divorced	2 (0.5)	1 (0.2)	2 (0.8)
**Household Income**			
Below $10,000	21 (5.0)	25 (5.9)	13 (5.5)
$10,000 to $19,999	20 (4.7)	40 (9.5)	11 (4.6)
$20,000 to $39,999	43 (10.2)	50 (11.8)	29 (12.2)
$40,000 to $59,999	56 (13.3)	65 (15.4)	27 (11.4)
$60,000 to $79,999	70 (16.6)	48 (11.4)	33 (13.9)
$80,000 and above	199 (47.2)	179 (42.4)	114 (48.1)
**Highest Level of Education**			
Less than high school	35 (8.3)	34 (8.0)	16 (6.8)
Completed high school	40 (9.5)	54 (12.8)	19 (8.0)
Some community college or technical school	40 (9.5)	31 (7.3)	24 (10.1)
Completed community college or technical school	93 (22.0)	92 (21.8)	41 (17.3)
Some university	39 (9.2)	39 (9.2)	20 (8.4)
Completed bachelor’s degree	122 (28.9)	107 (25.4)	77 (32.5)
Graduate degree	52 (12.3)	63 (14.9)	36 (15.2)
**Racial/Ethnic Background**			
White	316 (74.9)	291 (69.0)	174 (73.4)
Aboriginal	14 (3.3)	23 (5.5)	17 (7.2)
Black	13 (3.1)	4 (0.9)	3 (1.3)
Chinese	18 (4.3)	15 (3.6)	9 (3.8)
Filipino	18 (4.3)	27 (6.4)	4 (1.7)
Latin American	8 (1.9)	5 (1.2)	5 (2.1)
South Asian	13 (3.1)	7 (1.7)	6 (2.5)
Other	18 (4.3)	40 (9.5)	16 (6.8)
**Born in Canada**			
Yes	324 (76.8)	318 (75.4)	191 (80.6)
No	92 (21.8)	102 (24.2)	42 (17.7)
**Language Spoken Most Often at Home**			
English	352 (83.4)	352 (83.4)	205 (86.5)
French	8 (1.9)	5 (1.2)	1 (0.4)
Chinese	9 (2.1)	7 (1.7)	4 (1.7)
Tagalog (Filipino)	3 (0.7)	13 (3.1)	2 (0.8)
Other	32 (7.6)	24 (5.7)	13 (5.6)
Prenatal Care Provider**			
Family physician	254 (60.0)	253 (60.0)	149 (62.9)
Obstetrician	270 (64.0)	281 (66.6)	158 (66.7)
Midwife	46 (11.0)	39 (9.2)	27 (11.4)
Nurse practitioner	30 (7.0)	56 (13.3)	45 (19.0)
**Site of Prenatal Care**			
Private office	211 (50.0)	165 (39.1)	73 (30.8)
Clinic	175 (41.5)	201 (47.6)	87 (36.7)
Outpatient department of a hospital	28 (6.6)	42 (10.0)	47 (19.8)
Type of Delivery***			
Vaginal	289 (68.5)	318 (75.4)	154 (65.0)
Planned C-section	62 (14.7)	47 (11.1)	12 (5.1)
Unplanned C-section	71 (16.8)	55 (13.0)	28 (11.8)
Parity***			
Primipara	169 (40.0)	157 (37.2)	113 (48.3)
Multipara	239 (56.6)	248 (58.8)	103 (40.0)
**Maternal Health**			
Chronic health problem	49 (11.6)	37 (8.8)	37 (15.6)
Complication during pregnancy	104 (24.6)	100 (23.7)	39 (16.7)
Medical problem since delivery	20 (4.7)	18 (4.3)	21 (8.9)
Infant***			
Boy	224 (53.1)	194 (46.0)	87 (36.7)
Girl	198 (46.9)	227 (53.8)	106 (44.7)
**Variable**	**Mean (*SD*)**	**Mean (*SD*)**	**Mean (*SD*)**
Maternal age (years)	30.2 (5.3)	30.2 (5.1)	29.7 (4.8)
Gestational age at first prenatal care visit (weeks)	10.9 (9.0)	10.6 (5.8)	10.2 (5.4)
Gestational age at delivery (weeks)***	39.2 (1.4)	39.3 (2.0)	39.6 (1.2)
Birth weight of infant (grams)***	3406.3 (544.3)	3465.9 (496.3)	3506.8 (472.2)

^1^Missing responses were excluded from analyses.

*Halifax did not participate in Phase Five of the study.

**Percentages reported for prenatal care providers do not add to 100 as women were instructed to check off all that applied.

***In Phase Five, responses for these items are reported for Time 2 participants (n = 194 postpartum women).

1. Information Sharing: The 9 items within this factor focus on how prenatal care providers answer questions, keep information confidential, and ensure women understand reasons for tests and their results.

2. Anticipatory Guidance: The 11 items in this factor focus on women being given enough information to make decisions about their prenatal care and how their prenatal care providers prepare and give women options for their birth experience.

3. Sufficient Time: The 4 items within this factor focus on the time prenatal care providers spend addressing women’s questions and the time spent in an appointment.

4. Approachability: The 4 items in this factor address the health care provider’s approachability (e.g., woman was afraid to ask questions, felt like she was wasting prenatal care provider’s time).

5. Availability: The 5 items in this factor include knowing how to contact the prenatal care provider and how available the clinic/office staff or prenatal care provider are to respond to questions, concerns or needs.

6. Support and Respect: This factor has 12 items related to women being respected and supported by their prenatal care providers in regard to their concerns and decisions.

We used the Flesch-Kincaid Grade Level test, available in Microsoft Word, to assess the readability of the 46-item QPCQ. This test rates text on a U. S. school grade level, which is similar to the Canadian grade level system. The QPCQ had a Flesch-Kincaid grade level score of 8.7, which means that women with a grade 9 education can read and understand the items in the QPCQ.

### Phase four: validity and reliability testing

The final sample for Phase Four consisted of 422 women. Demographic characteristics of the participants are summarized in Table 2.

Confirmatory factor analysis verified and confirmed the presence of six factors, and all 46 items were therefore retained in the QPCQ. Refer to Table 3 for a list of the items loading on each factor. The factor (or subscale) means and standard deviations are presented in Table 4. Each subscale mean score was calculated by first reversing the scores of any reverse scored items in the subscale, then summing the scores for the items of the subscale and dividing the sum by the number of items. The QPCQ is a norm-referenced measure, in which an individual’s score takes on meaning when compared with the scores of others (e.g., in the same sample) [46]. Higher scores on the QPCQ and its subscales reflect a higher rating of quality of prenatal care. The mean scores for the factors ranged from 3.84 to 4.37 out of a total score of 5, indicating that women rated the quality of their prenatal care toward the higher end of the continuum. The factor “Anticipatory Guidance” had the lowest mean rating, while “Information Sharing” had the highest mean rating.

**Table 3:** Items loading on each factor, corrected item-total subscale correlations, and Cronbach’s alpha if item deleted from subscale

**Factor (Subscale) items**	**Corrected item-total subscale correlation**	**Cronbach’s alpha if item deleted from subscale**
**Factor 1: Information Sharing (9 items) Cronbach’s Alpha = .86**		
- I was given adequate information about prenatal tests and procedures	.60	.84
- I was always given honest answers to my questions	.56	.85
- Everyone involved in my prenatal care received the important information about me	.45	.86
- I was screened adequately for potential problems with my pregnancy	.47	.85
- The results of tests were explained to me in a way I could understand	.67	.83
- My prenatal care provider(s) gave straightforward answers to my questions	.70	.83
- My prenatal care provider(s) gave me enough information to make decisions for myself	.67	.83
- My prenatal care provider(s) kept my information confidential	.51	.85
- I fully understood the reasons for blood work and other tests my prenatal care provider(s) ordered for me	.66	.83
**Factor 2: Anticipatory Guidance (11 items) Cronbach’s Alpha = .85**		
- My prenatal care provider(s) gave me options for my birth experience	.55	.83
- I was given enough information to meet my needs about breast-feeding	.47	.84
- My prenatal care provider(s) prepared me for my birth experience	.57	.83
- My prenatal care provider(s) spent time talking with me about my expectations for labor and delivery	.61	.83
- I was given enough information about the safety of moderate exercise during pregnancy	.46	.84
- I received adequate information about my diet during pregnancy	.60	.83
- My prenatal care provider(s) was interested in how my pregnancy was affecting my life	.58	.83
- I was linked to programs in the community that were helpful to me	.41	.85
- I received adequate information about alcohol use during pregnancy	.39	.85
- I was given adequate information about depression in pregnancy	.58	.83
- My prenatal care provider(s) took time to ask about things that were important to me	.66	.83
**Factor 3: Sufficient Time (5 items) Cronbach’s Alpha = .81**		
- I had as much time with my prenatal care provider(s) as I needed	.54	.79
- My prenatal care provider(s) was rushed	.48	.84
- My prenatal care provider(s) always had time to answer my questions	.70	.75
- My prenatal care provider(s) made time for me to talk	.73	.73
- My prenatal care provider(s) took time to listen	.68	.75
**Factor 4: Approachability (4 items) Cronbach’s Alpha = .73**		
- My prenatal care provider(s) was abrupt with me	.50	.68
- I was rushed during my prenatal care visits	.49	.69
- My prenatal care provider(s) made me feel like I was wasting their time	.56	.65
- I was afraid to ask my prenatal care provider(s) questions	.55	.65
**Factor 5: Availability (5 items) Cronbach’s Alpha = .82**		
- I knew how to get in touch with my prenatal care provider(s)	.54	.80
- Someone in my prenatal care provider(s)’s office always returned my calls	.48	.82
- My prenatal care provider(s) was available when I had questions or concerns	.63	.77
- I could always reach someone in the office/clinic if I needed something	.71	.74
- I could reach my prenatal care provider(s) by phone when necessary	.68	.75
**Factor 6: Support and Respect (12 items) Cronbach’s Alpha = .93**		
- My prenatal care provider(s) respected me	.63	.93
- My prenatal care provider(s) respected my knowledge and experience	.63	.93
- My decisions were respected by my prenatal care provider(s)	.73	.92
- My prenatal care provider(s) was patient	.67	.93
- I was supported by my prenatal care provider(s) in doing what I felt was right for me	.71	.92
- My prenatal care provider(s) supported me	.75	.92
- My prenatal care provider(s) paid close attention when I was speaking	.70	.92
- My concerns were taken seriously	.71	.92
- I was in control of the decisions being made about my prenatal care	.69	.92
- My prenatal care provider(s) supported my decisions	.80	.92
- I was at ease with my prenatal care provider(s)	.68	.93
- My values and beliefs were respected by my prenatal care provider(s)	.69	.92

**Table 4:** QPCQ factor (or subscale) means and standard deviations (SD) from phase four (N = 422)

**Subscale**	**Mean (SD)**
Factor 1 – Information Sharing	4.37 (0.50)
Factor 2 – Anticipatory Guidance	3.84 (0.60)
Factor 3 – Sufficient Time	4.16 (0.65)
Factor 4 – Approachability	4.22 (0.71)
Factor 5 – Availability	4.18 (0.65)
Factor 6 – Support and Respect	4.35 (0.52)
Total QPCQ	4.19 (0.50)

A significant positive correlation between the QPCQ total score and the satisfaction subscale score of the PESPC provided additional support for construct validity (Pearson *r* = 0.81). Convergent validity was demonstrated by a significant positive correlation (*r* = 0.63) between the “Support and Respect” subscale of the QPCQ and the “Respectfulness/Emotional Support” subscale of the PIPC, and a significant positive correlation (*r* = 0.59) between the “Anticipatory Guidance” subscale of the QPCQ and the “Empowerment/Self-care” subscale of the PIPC.

Testing showed acceptable internal consistency reliability for the overall scale (Cronbach’s alpha = 0.96) and for the six subscales (ranging from 0.73-0.93). Refer to Table 3 for the results. Item-total scale correlation coefficients were positive, and the Cronbach’s alpha did not increase if any of the items were deleted, with the exception of one item, “My prenatal care provider was rushed,” showing a slight increase.

Of the 422 participants, 182 women (43%) completed the retest version of the QPCQ 5 to 14 days later and returned it by mail. The QPCQ demonstrated acceptable test-retest reliability (ICC = 0.88), indicating stability of the instrument on repeat administration.

### Phase five: temporal stability testing

Demographic characteristics of the participants in Phase Five (Time 1) are summarized in Table 2, and the sample size for each site and time period is shown in Table 5. There were 234 participants at Time 1, 194 at Time 2, and 158 at Time 3, demonstrating some attrition over time. There were no statistically significant differences in mean scores across time periods for the majority of the QPCQ subscales (Tables 6, 7, and 8). Although there was a significant difference in mean score for the Anticipatory Guidance subscale between Time 1 and 2 (*d* = 0.22) and between Time 1 and 3 (*d* = 0.17), and for the mean QPCQ score between Time 1 and 2 (*d* = 0.07), the differences in mean scores were small and deemed not to be clinically significant. The intra-class correlation coefficient (ICC) was also used to examine stability of the QPCQ subscale scores across the three time periods, and varied from 0.67 to 0.76 (Table 9). The ICC for the total QPCQ score was 0.81 (95% CI: 0.76-0.85).

**Table 5:** Number of participants per site for each time period in phase five of the study

**Recruitment site**	Before delivery QPCQ –T1*	After delivery QPCQ –T2*	4-6 week QPCQ –T3*	**Total matched T1/T2**	**Total matched T2/T3**	**Total matched T1/T2/T3**
	**n (%)**	**n (%)**	**n (%)**	**n (%)**	**n (%)**	**n (%)**
Vancouver	9 (4)	6 (3)	5 (2)	6 (3)	5 (2)	5 (2)
Calgary	79 (33)	77 (32)	65 (27)	74 (31)	64 (27)	62 (26)
Winnipeg	67 (28)	42 (18)	32 (14)	42 (18)	32 (14)	32 (14)
Hamilton	79 (33)	69 (29)	56 (24)	69 (29)	56 (24)	56 (24)
SUBTOTAL	234	194	158	191	157	155

*T1 = time one, T2 = time two, T3 = time three.

**Table 6:** Comparison of QPCQ subscale and total scores between Time 1 and Time 2 in Phase five, using paired t-test

**Subscale**	**N**	**Time 1**	**Time 2**	**p**
		**Late pregnancy**	**Early postpartum**	
		**Mean (SD)**	**Mean (SD)**	
Factor 1 –Information Sharing	191	4.27 (0.52)	4.29 (0.50)	0.41
Factor 2 – Anticipatory Guidance	191	3.55 (0.73)	3.77 (0.66)	<0.001
Factor 3 – Sufficient Time	191	4.09 (0.67)	4.10 (0.68)	0.69
Factor 4 – Approachability	191	4.24 (0.68)	4.25 (0.71)	0.92
Factor 5 – Availability	191	4.02 (0.63)	4.07 (0.66)	0.19
Factor 6 – Support and Respect	191	4.23 (0.55)	4.26 (0.58)	0.52
Total QPCQ	191	4.04 (0.53)	4.11 (0.52)	0.01

**Table 7:** Comparison of QPCQ subscale and total scores between Time 1 and Time 3 in Phase five, using paired t-test

**Subscale**	**N**	**Time 1**	**Time 3**	**p**
		**Late pregnancy**	**4-6 weeks postpartum**	
		**Mean (SD)**	**Mean (SD)**	
Factor 1 – Information Sharing	155	4.29 (0.45)	4.27 (0.44)	0.43
Factor 2 – Anticipatory Guidance	155	3.53 (0.70)	3.70 (0.67)	<0.001
Factor 3 – Sufficient Time	155	4.11 (0.64)	4.12 (0.56)	0.73
Factor 4 – Approachability	155	4.30 (0.60)	4.31 (0.61)	0.75
Factor 5 – Availability	155	4.02 (0.58)	4.04 (0.68)	0.70
Factor 6 – Support and Respect	155	4.25 (0.51)	4.25 (0.51)	0.97
Total QPCQ	155	4.05 (0.48)	4.09 (0.48)	0.12

**Table 8:** Comparison of QPCQ subscale and total scores between Time 2 and Time 3 in Phase five, using paired t-test

**Subscale**	**N**	**Time 2**	**Time 3**	**p**
		**Early postpartum**	**4-6 weeks postpartum**	
		**Mean (SD)**	**Mean (SD)**	
Factor 1 – Information Sharing	157	4.31 (0.44)	4.26 (0.44)	0.05
Factor 2 – Anticipatory Guidance	157	3.77 (0.64)	3.69 (0.67)	0.02
Factor 3 – Sufficient Time	157	4.14 (0.60)	4.12 (0.56)	0.47
Factor 4 – Approachability	157	4.31 (0.65)	4.31 (0.60)	0.99
Factor 5 – Availability	157	4.08 (0.60)	4.04 (0.68)	0.16
Factor 6 – Support and Respect	157	4.27 (0.54)	4.25 (0.50)	0.36
Total QPCQ	157	4.13 (0.47)	4.09 (0.48)	0.05

**Table 9:** Intra-class correlation coefficients for QPCQ subscales across three time points in Phase five

**Factor Name**	**Intra-class correlation coefficient**	**95% confidence interval**
1 – Information Sharing	0.75	0.69-0.80
2 – Anticipatory Guidance	0.76	0.71-0.81
3 – Sufficient Time	0.76	0.70-0.81
4 – Approachability	0.67	0.61-0.74
5 – Availability	0.76	0.71-0.81
6 – Support and Respect	0.74	0.69-0.79
Total score	0.81	0.76-0.85

## Discussion

Measurement of the quality of prenatal care is an essential step in more fully evaluating its effectiveness. We have developed a new instrument, the Quality of Prenatal Care Questionnaire (QPCQ), through a rigorous process of item generation and psychometric testing. The QPCQ was designed to be completed by women who received prenatal care, consistent with growing acknowledgement of the value of the consumer’s viewpoint in evaluating quality of health care [22,23,62,63]. The final 46-item version of the QPCQ demonstrated construct validity, as well as acceptable internal consistency and test-retest reliability. Having women complete the QPCQ before delivery, during their postpartum hospital stay, and again 4 to 6 weeks after delivery confirmed that women’s ratings of their quality of prenatal care did not change as a result of giving birth or between the early postpartum period and 4 to 6 weeks postpartum. These results suggest that the QCPQ can be administered to a woman after 36 weeks gestation and up to 6 weeks postpartum.

Exploratory factor analysis resulted in a six-factor solution for the QPCQ, with six factors retained in the confirmatory factor analysis. This indicates that the concept of quality of prenatal care is multidimensional and the instrument consists of six subscales [56]. In addition to the total QPCQ score, the score for each of the subscales can be examined separately. The derived factors made conceptual sense, and were consistent with the themes arising from our qualitative descriptive study [48]. The six subscales of the QPCQ measure both structure and process attributes of Donabedian’s model, with more emphasis on clinical and interpersonal processes of care. Although the initial draft of the QPCQ contained several items related to structure of prenatal care, many of these items were rated low on importance in Phase One and were subsequently deleted from the questionnaire (e.g., “The office/clinic was in a convenient location,” “The waiting area was crowded.”). This is consistent with Campbell’s viewpoint that structure is not a component of care “but the conduit through which care is delivered and received” [37]. As such, structure may influence the way in which care is provided and thus women’s assessment of quality. For example, having adequate funding, facilities and personnel may influence women’s responses to items in the “Sufficient Time” subscale (e.g., “I had as much time with my prenatal care provider as I needed”) and the “Availability” subscale (e.g., “I could always reach someone in the office/clinic if I needed something”). Items in the QPCQ “Information Sharing” and “Anticipatory Guidance” subscales primarily measured the clinical or technical processes of care, while items in the “Approachability” and “Support and Respect” subscales reflected interpersonal processes. Mean scores for the subscales ranged from 3.84 to 4.37, and indicated that women rated the quality of “Anticipatory Guidance” the lowest, and “Information Sharing” and “Support and Respect” the highest (Table 4). In the temporal stability testing phase, the Anticipatory Guidance subscale was the only one showing significant (although small) differences in mean scores over time, with both postpartum scores being higher than the prenatal score. Some of the Anticipatory Guidance items may be more accurately assessed by women in the postpartum period (e.g., “I was given enough information to meet my needs about breastfeeding”), possibly resulting in higher rating scores.

The subscales and items in the QPCQ measure components of quality of prenatal care identified by women as important in other qualitative studies [17-19] and an integrative review [64]. Wheatley and colleagues found that markers of quality prenatal care included the extent to which the provider listened carefully, showed respect, explained things, and spent enough time with the woman [18]. The main elements of quality of maternity care services identified in Goberna-Tricas’s study were technical expertise of the health professional, the human dimension of the relationship between the caregiver and the patient (interpersonal skill), and the structural aspects that determine the context in which the health care is provided [17]. Hildingsson and Thomas analyzed responses of 827 Swedish pregnant women to an open ended question in a survey, and grouped the findings into the following categories: technical aspects of care (being skilled and competent), psychological aspects of care (being a good listener, being supportive, treating the woman with respect), personal characteristics (not judging, not being rushed), health-related content and information (checking the baby’s health, providing information about physical and mental changes and breastfeeding), and structural aspects of provider visits (enough time during visits, continuity of care) [19]. The items in the QPCQ capture the majority of these aforementioned elements of quality of prenatal care.

### Strengths and limitations of the study

The QPCQ was developed taking into consideration effective prenatal care practices, the diversity of the Canadian population, and variations in the way prenatal care is delivered, and with input from both consumers and providers of care. The five study sites provided a broad cross-section of the childbearing population in Canada and its multicultural uniqueness. For instance, Winnipeg has a large and growing Aboriginal population, Vancouver has a high concentration of immigrants from East Asia, and Halifax serves a large rural population. Similarly, there are differences in the options for prenatal care available to women across the five study sites. Midwifery care was not regulated or integrated into the health care system in Nova Scotia at the time of this study, but was more widely available to women living in certain areas of Ontario, such as Hamilton, and other provinces where midwifery was a regulated profession. In some provinces, obstetricians were the most common provider of prenatal care (e.g., Ontario) compared to family physicians in others (e.g., British Columbia) [65]. Finally, some prenatal programs had integrated additional or substitutive prenatal care through nurse specialists and nurse practitioners [66]. The study protocol thereby ensured the development of an instrument that captured core elements of quality applicable to the Canadian population as a whole under a system of universal health care.

Our study also has limitations. The QPCQ was developed in the context of the Canadian health care system, so its applicability to health care systems, prenatal care provision, or populations that are substantively different will need to be assessed prior to widespread use. The instrument was intended to be applicable to all pregnant women; therefore the items may not fully capture all elements of quality in specific situations, such as care provided to women with a complicated or high risk pregnancy. The QPCQ reflects the woman’s perception of the quality of prenatal care she received; further research is needed to determine the congruence between the woman’s assessment of quality and the extent to which the care she received conformed to guidelines for prenatal care using methods such as chart audits. The relatively high mean scores found among some of the QPCQ subscales may be a reflection of selection bias incurred as a result of using a convenience sample, in that women who agreed to participate in the study may have viewed the quality of their care more positively than women who declined participation. In addition, the response rate for completion of the retest version of the QPCQ was relatively low (43%), although the number of respondents (n = 182) exceeded the minimum sample size of 79 estimated as needed in the sample size calculation.

Finally, we acknowledge there are competing views regarding use of non-parametric versus parametric statistics to analyze Likert scales [67,68]. Although individual Likert *items* are ordinal in character, we support the position that Likert *scales* (collections of Likert items) produce interval data, and that it is appropriate to summarize the ratings generated from Likert scales using means and standard deviations, and to use parametric statistics to analyze the scales [68]. Health care providers may find it helpful to examine the rank order of (dis)agreement for individual items on the QPCQ to identify specific aspects of prenatal care in need of quality improvement. However, for research using the QPCQ, we agree with Carifio and Perla’s view that treating the data from Likert scales as interval in character permits “more powerful and nuanced analyses” [68].

### Recommendations for future research

This valid and reliable instrument can now be used as an outcome measure to evaluate quality of prenatal care, to identify predictors of quality of prenatal care, to compare and contrast quality of prenatal care across regions, populations, and types of health care providers and service delivery models, and to assess the relationship between quality of care and a variety of maternal and infant health outcomes. The outcomes studied should not be limited to gestational age and birth weight, but rather encompass a range of health status and behavioral indicators. As noted by Alexander and Kotelchuck, “there are several other perinatal outcomes that may be modified by prenatal care” [1]. Rosenberg has suggested that attention should be given to studying the effect of optimal prenatal care on maternal self-esteem, attachment, connections to both the health care system and social services, and maternal physical and mental health [69]. Other appropriate outcomes include postnatal health status of mother and infant, the adoption and maintenance of healthy behaviors, disclosure of sensitive concerns, postpartum behaviors, maternal and infant health care utilization, and infant injury and disease rates [1]. The relationship between quality of care and a variety of outcomes may have implications for allocation of resources, program planning, and policy development. With a valid and reliable QPCQ, researchers and decision makers will be well positioned to collect evidence that can be used to design and refine programs to improve women’s experiences and enhance perinatal outcomes.

## Conclusions

The QPCQ is a new self-report instrument that measures overall quality of prenatal care, and quality of care for six factors or subscales. Following a rigorous process of development and psychometric testing, the QPCQ has been shown to demonstrate construct validity, internal consistency reliability, and test-retest reliability. This valid and reliable instrument will be useful in future research to evaluate women’s perceptions of quality of prenatal care, to compare quality of care across regions, populations, types of health care provider, and service delivery models, and to assess the relationship between quality of care and a variety of maternal and infant health outcomes.

## Abbreviations

QPCQ: Quality of Prenatal Care Questionnaire; CTP: Content and timing of care in pregnancy tool; PESPC: Patient Expectations and Satisfaction with Prenatal Care instrument; PIPC: Prenatal Interpersonal Processes of Care instrument; ICC: Intra-class correlation coefficient; RBD: Randomized block design.

## Competing interests

The authors declare that they have no competing interests.

## Authors’ contributions

WAS and MIH wrote the grant application, directed the implementation of the study protocol, and had overall responsibility for the research. All authors contributed to conception and design of the study, and interpretation of the results, with input from the collaborators. AB coordinated the study. MIH, WAS, ST, PAJ, and DCY supervised participant recruitment in their respective sites. NA-D performed data analysis, assisted by AB. MIH drafted the manuscript. All authors provided feedback on the draft manuscript, and read and approved the final manuscript.

## Pre-publication history

The pre-publication history for this paper can be accessed here:

http://www.biomedcentral.com/1471-2393/14/188/prepub
